# An efficient synthesis of novel pyrano[2,3-*d*]- and furopyrano[2,3-*d*]pyrimidines via indium-catalyzed multi-component domino reaction

**DOI:** 10.1186/1860-5397-2-11

**Published:** 2006-06-13

**Authors:** Dipak Prajapati, Mukut Gohain

**Affiliations:** 1 Department of Medicinal Chemistry, Regional Research Laboratory, Jorhat 785006, Assam, India

## Abstract

Various novel pyrano [2,3-*d*]pyrimidines **5** and furopyrano [2,3-*d*]pyrimidines **7** were synthesized in 80–99% yields via a multicomponent domino Knoevenagel/hetero-Diels-Alder reaction of 1,3-dimethyl barbituric acid with an aromatic aldehyde and ethyl vinyl ether/2,3-dihydrofuran in presence of 1 mol% of indium(III) chloride. The reaction also proceeds in aqueous media without using any catalyst, but the yield is comparatively less (65–70%).

## Introduction

**Scheme 1:**
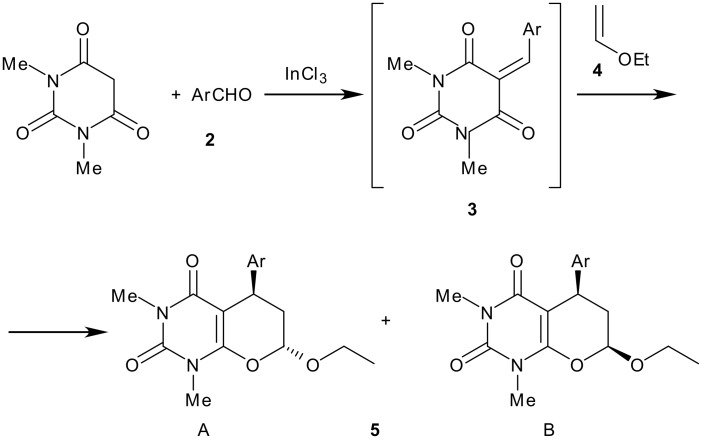
Reagents and conditions: i) 1 mol% InCl_3_, acetonitrile:water (3:1)

The emergence of indium(III) compounds as efficient Lewis acid catalysts presents new and exciting opportunities for organoindium chemistry. [1–2] It was found that the low reactivity of trivalent organoindium reagents can be increased by complex formation with organolithium compounds.[3] The tetra-organo-indates thus prepared are sufficiently reactive to take part in reactions at ambient temperature.[3] Moreover, indium metal[4] has been found to be an effective reducing agent and indium(III) halide or its complexes act[5] as an efficient, moisture compatable Lewis acid catalysts in Mukaiyama Aldol reactions, Friedel-Crafts acylations,[6] Pavarob reactions[7] and Diels-Alder reactions[8] in water. Since the work of Loh,[8] indium halide has also been shown to be an effective Lewis acid catalyst for various reactions in aqueous media. [9–10] However, its use in the hetero Diels-Alder reaction of α,β-ethylenic ketones and ethyl vinyl ether or 2,3-dihydrofuran has remained unexplored.[11] Herein, we report the first example of indium(III) chloride catalysed synthesis of fused pyrimidine derivatives via a multicomponent domino Knoevenagel hetero Diels-Alder reaction. The reaction proceeds efficiently at ambient temperature in excellent yields (Scheme 1).

Pyrimidine derivatives continue to be of great interest due to their wide range of biological activities.[12] Preparation of naturally occuring complex molecules containing a uracil ring pose significant synthetic challenges.[13] The development of clinically useful anticancer (5-fluorouracil)[14] and antiviral drugs (AZT, DDC, DDI, BVDU) [15–17] has renewed interest in the synthetic manipulation of uracils.[18] The synthesis of furopyrimidines has received little attention and only few procedures have been reported in the literature, [19–21] most of which rely on multi-step reactions with yields being low. [22–23] The furo [2,3-*d*]pyrimidine derivatives act as useful sedatives, antihistamines, diuretics, muscle relaxants and antiulcer agents. Furthermore, pyrano [2,3-*d*]pyrimidines also represent broad classes of annelated uracils. A number of compounds having these systems are synthesized with diverse pharmacological activity. [24–25] For the preparation of these complex molecules, there has been remarkable interest in the synthetic manipulations of uracils.[26] Also the synthetic exploitation of the nucleophilic double bond of uracil is an undeveloped field in view of a great variety of potential products. [27–29] Although a variety of routes for the synthesis of these compounds have been appeared in the literature, [30–33] the majority of them involve a number of steps, drastic conditions, long reaction time and low yields. Thus new routes for the synthesis of these molecules have attracted considerable attention in search for a rapid entry to these heterocycles and their diverse biological properties. In search of an efficient method and in continuation to our studies on uracil analogues [34–35], we have investigated a new, simple and efficient synthesis of novel fused pyrimidines based on inverse electron-demand Diels-Alder reaction using ethyl vinyl ether/dihydrofuran and α,β-ethylenic ketones formed in *situ* as heterodienes in presence of 1 mol% of indium(III) trichloride (Scheme 1). This type of cycloaddition using an amino aldehyde and a benzyl enol ether in presence of ethylene diammonium acetate and triethyl orthoformate has been reported earlier but this method has its own merits and limitations.[36]

## Results and Discussions

On treatment of 1,3-dimethylbarbituric acid **1**, 4-nitrobenzaldehyde **2a**, ethyl vinyl ether **4** in acetonitrile:water (3:1) and in presence of 1 mol% indium(III) trichloride, a one-pot three-component reaction proceeded spontaneously at room temperature. After completion and usual work-up the corresponding pyrano [2,3-*d*]pyrimidine derivative **5a** was obtained in 99% yields as a mixture of diastereomers. The structure of the compound was confirmed as **5a** from the spectroscopic data and elemental analyses.

The NMR spectra showed the absence of the methylene proton of the 1,3-dimethylbarbituric acid and the presence of a proton at δ 5.46 or at δ 5.25. The configuration of the pyrano [2,3-*d*]pyrimidine **5** was determined to be a mixture of *cis* and *trans* compounds, where the *trans* diastereomers are always predominant; in most cases they were separated by silica gel chromatography. For, example, elution of **5a** with hexane/ethylacetate (30:10 v/v) afforded first the *trans* diastereomer as a solid mp 170–72°C and then the minor *cis* diastereomer as a crystalline compound mp 153–55°C in the ratio 75:25. The NMR spectra of diastereomers A (*trans*) and B (*cis*) of compound **5a** (for example) exhibited a resonance as a doublet of doublets attributable to the anomeric proton at δ = 5.46 (*J* = 3.0, and 6.3 Hz) for the diastereomer A and at δ = 5.25 (*J* = 2.1 and 4.5 Hz) for the diastereomer B, suggesting that the ethoxy substituent occupies a pseudoaxial position within the dihydropyran ring for the A diastereomer. The benzylic proton appeared as a triplet at δ = 4.15 and 4.19 for the diastereomer A and B with an apparent coupling constant *J* = 7.5 and 6.6 Hz respectively. Analysis of the resonances of the equatorial and axial hydrogens located on the neighbouring methylene group of diastereomers A or B indicated, after spin decoupling, that the coupling constant are *J* = 7.0 and 6.1 Hz respectively for diastereomer A. The configuration of diastereomer A may therefore be assigned as *trans* and *cis* for the diasteromer B. The formation of these heterocycles **5** can be rationalized by initial formation of a conjugated electron-deficient heterodiene by standard Knoevenagel condensation of the aldehyde **2** with the 1,3-dimethylbarbituric acid,[37] which is highly activated due to the presence of the electron withdrawing group. It can, therefore, react with the ethyl vinyl ether at room temperature, to provide the cycloadduct **5** in a hetero-Diels-Alder reaction with inverse electron-demand. [38–40] Similarly, several aromatic aldehydes were reacted well to give the corresponding pyrano [2,3-*d*]pyrimidines in excellent yields ('see Supporting Information File 1'). However, the reaction did not proceed with aliphatic or heterocyclic aldehydes. The reaction is also effective when 1 mol% of scandium or ytterbium triflate was used as catalyst but indium chloride gives better results (Table 1). In a control experiment, the reaction also proceeded without the use of any catalysts, but it yields the compound **5** in a (1:1) mixture of diastereomers in 65–70% yields (Table 1).

**Table 1:** Inverse electron-demand hetero-Diels-Alder reaction for the synthesis of pyrano [2,3-*d*]pyrimidines 5 and furopyrano [2,3-*d*]pyrimidines **7**.

Products	Ar	Catalyst	Reaction time (h)	Yield (%)^a^ (cis:trans)	Mp (°C)^b^	
					A (trans)	B (cis)

**5a**	*4*-NO_2_-C_6_H_4_-	InCl_3_	2.5	99 (25:75)	170–72	153–55
**5b**	C_6_H_5_	InCl_3_	3.5	95 (30:70)	155–57	148–50
**5c**	*4-*Cl-C_6_H_4_-	InCl_3_	2.5	95 (30:70)	174–75	154–55
**5d**	*4*-CH_3_-C_6_H_4_	InCl_3_	3.5	90 (30:70)	165–68	146–48
**5e**	*4*-CH_3_O-C_6_H_4_	InCl_3_	3.5	90 (30:70)	198–99	189–90
**5a**	*4*-NO_2_-C_6_H_4_-	Sc(OTf)_3_	3.0	85 (45:55)	168–72	153–55
**5b**	C_6_H_5_	Sc(OTf)_3_	3.5	80 (35:65)	155–57	148–50
**5c**	*4-*Cl-C_6_H_4_-	Sc(OTf)_3_	3.0	80 (45:55)	174–75	154–55
**5d**	*4*-CH_3_-C_6_H_4_	Sc(OTf)_3_	3.5	80 (45:55)	165–68	146–48
**5e**	*4*-CH_3_O-C_6_H_4_	Sc(OTf)_3_	3.5	82 (25:75)	198–99	189–90
**5a**	*4*-NO_2_-C_6_H_4_-	--	4.0	70 (50:50)	168–72	153–55
**5b**	C_6_H_5_	--	4.5	65 (50:50)	155–57	148–50
**5d**	*4*-CH_3_-C_6_H_4_	--	4.5	70 (50:50)	165–66	146–48
**7a**	*4*-NO_2_-C_6_H_4_-	InCl_3_	8.0	80	145–47	--
**7b**	C_6_H_5_	InCl_3_	10	85	156–57	--
**7c**	*4-*Cl-C_6_H_4_-	InCl_3_	8.0	90	178–80	--
**7d**	*4*-CH_3_-C_6_H_4_	InCl_3_	8.0	80	156–57	--
**7e**	*4*-CH_3_O-C_6_H_4_	InCl_3_	10	82	153–54	--

^a^Isolated yields. ^b^All products were characterized by ^1^H NMR IR and mass spectra.

**Scheme 2:**
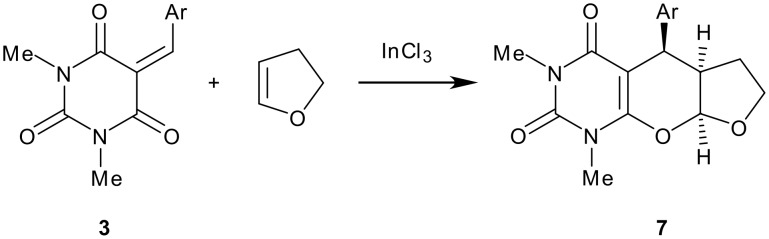
Reagents and conditions: i) 1 mol% InCl_3_, room temperature

To further investigate the synthetic scope of this cycloaddition reaction, we reacted 2,3-dihydrofuran with 1,3-dimethylbarbituric acid and an aromatic aldehyde at room temperature in presence of 1 mol% of indium(III) trichloride under similar conditions. The reaction after usual work-up gave the corresponding furopyrano [2,3-*d*]pyrimidine **7** in excellent yields. Knoevenagel condensation of the aromatic aldehyde with 1,3-dimethylbarbuturic acid occurs first with the formation of an electron-deficient, sterically fixed 1-oxo-1,3-butadiene **3** which then reacts with 2,3-dihydrofuran in a Diels-Alder reaction with inverse-electron demand[13] to afford furopyrano [2,3-*d*]pyrimidines **7** (Scheme 2). The structure of the compound **7** thus obtained was assigned on the basis of its elemental and spectral analyses ('see Supporting Information File 2'). The ^1^H NMR shows the absence of methylene proton of barbutric acid and the presence of a proton at δ 5.75 and two methyl groups of the cycloadducts **7** at δ = 3.25 (s, 3H, NMe), 3.48 (s, 3H, NMe) ppm. To generalize this reaction we reacted various substituted aromatic aldehydes and isolated the corresponding furopyrano [2,3-*d*]pyrimidines in 80–90% yields. In all cases the reactions proceeded smoothly at ambient temperature with high selectivity. The tetrahydrofuran ring is *cis* fused as depicted by the coupling constant of the protons at δ 5.75 *J* = 6 Hz and δ 4.48 *J* = 5.4 Hz. Several examples illustrating this novel and rapid procedure for the synthesis of fused pyrimidines are summarized in the Table 1. Remarkably, this Diels-Alder reaction was also proceeded similarly when 5-benzylidene barbituric acid was employed directly with 2,3-dihydrofuran in presence of 1 mol% of indium(III) chloride. The corresponding furopyrano [2,3-*d*]pyrimidine derivatives **7** were obtained in almost comparable yields. The structure of all the products thus obtained were characterized by elemental and spectral analyses.

## Conclusion

In conclusion, we have shown that the multicomponent domino Knoevenagel condensation/hetero-Diels-Alder reaction of aromatic aldehydes with 1,3-dimethylbarbituric acid and ethyl vinyl ether or dihydrofuran catalysed by InCl_3_ is a powerful and efficient method for the synthesis of novel fused pyrimidines of biological significance. In addition to its efficiency, simplicity and milder reaction conditions this method provides excellent yields of products with selectivity, which makes it a useful process for the synthesis of *cis*-fused furopyrano [2,3-*d*]pyrimidines and pyrano [2,3-*d*]pyrimidines.

## Supporting Information

File 1 contains full experimental data

File 2 contains supplementary information of compounds **5** &**7**.
