# Supplementary material for: An efficient synthesis of novel pyrano[2,3-d]- and furopyrano[2,3-d]pyrimidines via indium-catalyzed multi-component domino reaction
Source: Beilstein J Org Chem. 2006 Jun 13;2:11. doi: 10.1186/1860-5397-2-11 (PMC1525172; doi:10.1186/1860-5397-2-11)
Supplement: File 1 — contains full experimental data [file Beilstein_J_Org_Chem-02-11-s001.doc]

**An efficient synthesis of novel pyrano[2,3-*d*]- and furopyrano[2,3-*d*]pyrimidines via indium-catalyzed multi-component domino reaction**

Dipak Prajapati,*and Mukut Gohain

Experimental Section

Synthesis of pyrano[2,3-*d*]pyrimidines and furopyrano[2,3-*d*]pyrimidines in presence of indium(III) chloride: Typically, to a solution of N,N-dimethylbarbituric acid (1.60 g, 10 mmol) in 15 ml of acetonitrile:water (3:1), added 4-nitrobenzaldehyde (1.50 g, 10 mmol), ethyl vinyl ether (1.50 g, 20 mmol) and catalytic amount of indium trichloride (0.022 g, 0.1 mmol) and the resulting mixture was stirred at room temperature for 2.5 h. After completion of the reaction (monitiored by tlc), acetonitrile was evaporated in a rotary evaporator. The residue thus obtained was treated with cold water (10 ml) and extracted with ethylacetate (3x20 ml). The combined organic phases were dried over anhydrous sodium sulphate, evaporated and then subjected to column chromatography using hexane:ethylacetate (3:1) as the eluent to afford the corresponding pyrano[2,3-d]pyrimidine 5a in 99% yield, the isomers are 75:25 ratio mp of *trans* A isomer is 170-172 ^o^C (decomp) and the other *cis* B is 153-155 ^o^C (decomp). 5aA:IR (KBr, cm^-1^): 1643,1705,1748. MS m/z 361 (M^+^). ^1^H NMR (300 MHz, CDCl_3_) δ 1.09 (t, *J* = 6.9 Hz, 3H, CH_3_), 2.21 (td, *J* = 4, 11 Hz, 1H, CH_2_), 2.38 (ddd, *J* = 2.7, 5.1, 12.3 Hz, 1H, CH_2_), 3.29 (s, 3H, NMe), 3.45 (s, 3H, NMe), 3.55 (m, 1H, OCH_2_), 3.82 (m, 1H, OCH_2_), 4.15 (t, *J* = 7.5 Hz, 1H, CHPh), 5.46 (dd, *J* = 3.0, 6.3 Hz, 1H, CHO), 7.37 (d, *J* = 8.7 Hz, 2H, ArH), 8.09 (d, *J* = 8.7 Hz, 2H, ArH). ^13^C NMR δc 15.01, 27.95, 28.77, 33.88, 35.90, 66.02, 88.16, 100.68, 123.98, 128.08, 146.69, 151.14, 151.79, 155.48, 161.96. Anal calcd. for C_17_H_19_N_3_O_6_: C, 56.50; H, 5.26; N, 11.63. Found: C, 56.41; H, 5.14; N, 11.72. 5aB ^1^H NMR (300 MHz, CDCl_3_) δ 1.22 (t, *J* = 6.9 Hz, 3H, CH_3_), 2.07 (ddd, J = 2.4, 4.8, 7.0 Hz, 1H, CH_2_), 2.32 (m, 1H, CH_2_), 3.24 (s, 3H, NMe), 3.42 (s, 3H, NMe), 3.70 (m, 1H, OCH_2_), 3.95 (m, 1H, OCH_2_), 4.19 (t, *J* = 6.6, 14 Hz, 1H, CHPh), 5.25 (dd, *J* = 2.1, 4.5 Hz, 1H, CHO), 7.34 (m, 2H, ArH), 8.13(d, 2H, *J* = 8.7 Hz, ArH). ^13^C NMR δc 14.81, 28.03, 28.79, 33.75, 34.56, 65.57, 88.08, 95.45, 95.48, 101.62, 123.35, 128.35, 146.39, 151.14, 15159, 155.32, 160.09, 162,18. Similarly other pyrano[2,3-*d*]pyrimidines 4b-e and furopyrano[2,3-*d*]pyrimidine derivatives 7a-e were synthesized from the reactions of barbituric acid 1, aldehyde 2 and ethyl vinyl ether/2,3-dihydrofuran and the structures were confirmed from their spectroscopic data and elemental analyses. 7c: IR (KBr, cm^-1^) : 1647,1688,1709. MS m/z 348 (M^+^). ^1^H NMR (300 MHz, CDCl_3_) δ 1.25 (m, 1H, 2-H), 1.75 (m, 1H, 2-H), 2.85 (m, 1H, 5-H), 3.25 (s, 3H, NMe), 3.48 (s, 3H, NMe), 3.90 (m, 1H, 1-H), 4.20 (m, 1H, 1-H), 4.48 (d, *J* = 5.5 Hz, 1H, ), 5.75 (d, *J* = 5.8 Hz, 1H), 7.04-7.48 (m, 4H, Aromatics). ^13^C NMR δc 25.70, 27.71, 28.32, 29.31, 36.91, 44.68, 45.94, 69.23, 69.46, 103.19, 104.43, 128.44, 128.80, 129.26, 13975. Anal calcd. for C_17_H_17_N_2_O_4_Cl: C, 58.62; H, 4.88; N, 8.05. Found: C, 58.71; H, 4.76; N, 8.13.

Synthesis of pyrano[2,3-d]pyrimidines without using any catalysts: To a solution of N,N-dimethylbarbituric acid (1.60 g, 10 mmol) in 15 ml of acetonitrile:water (3:1), added 4-nitrobenzaldehyde (1.50 g, 10 mmol) and ethyl vinyl ether (1.50 g, 20 mmol) and the resulting mixture was stirred at room temperature for 3 h. After completion of the reaction (monitiored by tlc), acetonitrile was distilled in a rotary evaporator. The residue thus obtained was treated with cold water (10 ml) and extracted with ethylacetate (3x20 ml). The combined organic phases were dried over anhydrous sodium sulphate, evaporated and then subjected to column chromatography using hexane:ethylacetate (3:1) as the eluent to afford the corresponding pyrano[2,3-*d*]pyrimidine in 70% yield, the isomers are 50:50 ratio. Similarly some other pyrano[2,3-d]pyrimidine derivatives were synthesized from the reactions of barbituric acid 1, aldehyde 2 and 2,3-dihydrofuran 6 in 65-70% yields and the structures were confirmed from their spectroscopic data and elemental analyses.
