# Supplementary material for: An efficient synthesis of novel pyrano[2,3-d]- and furopyrano[2,3-d]pyrimidines via indium-catalyzed multi-component domino reaction
Source: Beilstein J Org Chem. 2006 Jun 13;2:11. doi: 10.1186/1860-5397-2-11 (PMC1525172; doi:10.1186/1860-5397-2-11)
Supplement: File 2 — contains supplementary information of compounds 5 &7. [file Beilstein_J_Org_Chem-02-11-s002.doc]

**An efficient synthesis of novel pyrano[2,3-*d*]- and furopyrano[2,3-*d*]pyrimidines via indium-catalyzed multi-component domino reaction**

Dipak Prajapati,*and Mukut Gohain

**Supplementary Information**:

**5bA**:IR (KBr, cm^-1^): 1619, 1669,1711, MS m/z 316 (M^+^). ^1^H NMR (300 MHz, CDCl_3_) δ 1.30 (t, *J* = 7.0 Hz, 3H, CH_3_), 2.06 (m, 1H, CH_2_), 2.30 (m, 1H, CH_2_), 3.32 (s, 3H, NMe), 3.48 (s, 3H, NMe), 3.74 (m, 1H, OCH_2_), 3.92 (m, 1H, OCH_2_), 4.36 (t, *J* = 6.8, 14 Hz, 1H, CHPh), 5.32 (dd, *J* = 2.2, 4.5 Hz, 1H, CHO), 7.26-8.10 (d, 5H, ArH). ^13^C NMR δc 15.20, 28.12, 28.76, 33.92, 35.88, 66.10, 88.12, 100.72, 124.08, 128.06, 146.66, 150.98, 151.82, 155.52, 162.06. Anal calcd. for C_17_H_20_N_2_O_4_: C, 64.56; H, 6.33; N, 8.86. Found: C, 64.64; H, 6.24; N, 8.95. **5bB** ^1^H NMR (300 MHz, CDCl_3_) δ 1.12 (t, *J* = 7.0 Hz, 3H, CH_3_), 2.20 (m, 1H, CH_2_), 2.32 (m,1H, CH_2_), 3.36 (s, 3H, NMe), 3.48 (s, 3H, NMe), 3.62 (m, 1H, OCH_2_), 3.88 (m, 1H, OCH_2_), 4.25 (dd, *J* = 7.2, 4.4 Hz, 1H, CHPh), 5.50 (dd, *J* = 6.3, 3.2 Hz, 1H, CHO), 7.28 (d, 2H, *J* = 8.6 Hz, 1H, ArH), 8.02 (d, 2H, *J* = 8.6 Hz, ArH). ^13^C NMR δc 14.92, 28.10, 28.84, 34.02, 34.62, 65.66, 88.10, 95.52, 96.02, 101.72, 123.45, 128.38, 146.32, 151.20, 151.65, 155.36, 162,20.

**5cA**:IR (KBr, cm^-1^): 1645,1702,1747. MS m/z 350 (M^+^). ^1^H NMR (300 MHz, CDCl_3_) δ 1.28 (t, 3H, CH_3_), 2.10 (m, 1H, CH_2_), 2.36 (m, 1H, CH_2_), 3.28 (s, 3H, NMe), 3.54 (s, 3H, NMe), 3.72 (m, 1H, OCH_2_), 3.98 (m, 1H, OCH_2_), 4.30 (t, *J* = 6.6, 14 Hz, 1H, CHPh), 5.46 (dd, *J* = 2.2, 4.5 Hz, 1H, CHO), 7.22 (d, 2H, *J* = 8.6 Hz, ArH), 8.06 (d, 2H, *J* = 8.6 Hz, ArH). Anal calcd. for C_17_H_19_N_2_O_4_Cl: C, 58.29; H, 5.43; N, 8.00. Found: C, 58.37; H, 5.36; N, 8.11. **5cB** ^1^H NMR (300 MHz, CDCl_3_) δ 1.18 (t, 3H, CH_3_), 2.32 (m, 1H, CH_2_), 2.44 (m,1H, CH_2_), 3.30 (s, 3H, NMe), 3.52 (s, 3H, NMe), 3.64 (m, 1H, OCH_2_), 3.86 (m, 1H, OCH_2_), 4.28 (dd, *J* = 7.4, 4.4 Hz, 1H, CHPh), 5.64 (dd, *J* = 6.4, 3.3 Hz, 1H, CHO), 7.22 (d, 2H, *J* = 8.6 Hz, 1H, ArH), 7.98 (d, 2H, *J* = 8.6 Hz, ArH).

**5dA**:IR (KBr, cm^-1^): 1647,1705. MS m/z 330 (M^+^). ^1^H NMR (300 MHz, CDCl_3_) δ 1.12 (t, 3H, CH_3_), 2.08 (s, 3H, CH_3_), 2.32 (m, 1H, CH_2_), 2.48 (m, 1H, CH_2_), 3.44 (s, 3H, NMe), 3.68 (s, 3H, NMe), 3.76 (m, 1H, OCH_2_), 4.02 (m, 1H, OCH_2_), 4.38 (t, *J* = 6.7, 14 Hz, 1H, CHPh), 5.62 (dd, *J* = 2.2, 4.5 Hz, 1H, CHO), 7.10 (d, 2H, *J* = 8.7 Hz, ArH), 7.82 (d, 2H, *J* = 8.7 Hz, ArH). Anal calcd. for C_18_H_22_N_2_O_4_: C, 65.45; H, 6.67; N, 8.48. Found: C, 65.56; H, 6.59; N, 8.46. **5dB** ^1^H NMR (300 MHz, CDCl_3_) δ 0.98 (t, 3H, CH_3_), 1.98 (s, 3H, CH_3_), 2.18 (m, 1H, CH_2_), 2.30 (m,1H, CH_2_), 3.26 (s, 3H, NMe), 3.48 (s, 3H, NMe), 3.66 (m, 1H, OCH_2_), 3.86 (m, 1H, OCH_2_), 4.35 (dd, *J* = 7.5, 4.2 Hz, 1H, CHPh), 5.48 (dd, *J* = 6.2, 3.2 Hz, 1H, CHO), 7.12 (d, 2H, *J* = 8.6 Hz, 1H, ArH), 7.92 (d, 2H, *J* = 8.6 Hz, ArH).

**5eA**:IR (KBr, cm^-1^): 1647, 1678, 1747. MS m/z 347 (M^+^). ^1^H NMR (300 MHz, CDCl_3_) δ 1.20 (t, 3H, CH_3_), 1.96 (m, 1H, CH_2_), 2.22 (m, 1H, CH_2_), 3.28 (s, 3H, NMe), 3.54 (s, 3H, NMe), 3.60 (m, 1H, OCH_2_), 3.72 (s, 3H, OCH_3_), 3,98 (m, 1H, OCH_2_), 4.35 (t, *J* = 6.6, 14 Hz, 1H, CHPh), 5.36 (dd, *J* = 2.2, 4.5 Hz, 1H, CHO), 7.26 (d, 2H, *J* = 8.7 Hz, ArH), 8.02 (d, 2H, *J* = 8.7 Hz, ArH). Anal calcd. for C_18_H_22_N_2_O_5_: C, 62.43; H, 6.36; N, 8.09. Found: C, 62.49; H, 6.42; N, 7.99. **5dB** ^1^H NMR (300 MHz, CDCl_3_) δ 1.02 (t, 3H, CH_3_), 2.24 (m, 1H, CH_2_), 2.38 (m, 1H, CH_2_), 3.24 (s, 3H, NMe), 3.42 (s, 3H, NMe), 3.62 (m, 1H, OCH_2_), 3.78 (s, 3H, OCH_3_), 3.92 (m, 1H, OCH_2_), 4.26 (dd, *J* = 7.5, 4.2 Hz, 1H, CHPh), 5.54 (dd, *J* = 6.3, 3.3 Hz, 1H, CHO), 7.08 (d, 2H, *J* = 8.7 Hz, ArH), 8.06 (d, 2H, *J* = 8.7 Hz, ArH).

7a: IR (KBr, cm^-1^) : 1631,1669,1701. MS m/z 359 (M^+^). ^1^H NMR (300 MHz, CDCl_3_) δ 1.18 (m, 1H, 2-H), 1.65 (m, 1H, 2-H), 2.72 (m, 1H, 3-H), 3.22 (s, 3H, NMe), 3.40 (s, 3H, NMe), 3.94 (m, 1H, 1-H), 4.15 (m, 1H, 1-H), 4.54 (d, *J* = 5.4 Hz, 1H, ), 5.88 (d, *J* = 6.0 Hz, 1H), 6.92-7.38 (m, 4H, ArH). ^13^C NMR δc 24.12, 26.98, 28.26, 30.02, 37.10, 45.06, 46.12, 69.44, 69.92, 102.98, 104.62, 128.32, 128.98, 129.36, 140.10. Anal calcd. for C_17_H_17_N_3_O_6_: C, 56.82; H, 4.74; N, 11.70. Found: C, 56.90; H, 4.76; N, 11.81.

7b: IR (KBr, cm^-1^) : 1645,1675,1705. MS m/z 314 (M^+^). ^1^H NMR (300 MHz, CDCl_3_) δ 1.22 (m, 1H, 2-H), 1.58 (m, 1H, 2-H), 2.66 (m, 1H, 3-H), 3.18 (s, 3H, NMe), 3.42 (s, 3H, NMe), 3.86 (m, 1H, 1-H), 4.12 (m, 1H, 1-H), 4.36 (d, *J* = 5.5 Hz, 1H, ), 5.86(d, *J* = 6.0 Hz, 1H), 7.00-7.45 (m, 5H, ArH). Anal calcd. for C_17_H_18_N_2_O_4_: C, 64.96; H, 5.73; N, 8.92. Found: C, 64.88; H, 5.82; N, 8.83.

7d: IR (KBr, cm^-1^) : 1645,1694,1706. MS m/z 329 (M^+^). ^1^H NMR (300 MHz, CDCl_3_) δ 1.24 (m, 1H, 2-H), 1.82 (m, 1H, 2-H), 2.93 (m, 1H, 3-H), 3.32 (s, 3H, NMe), 3.56 (s, 3H, NMe), 3.98 (m, 1H, 1-H), 4.25 (m, 1H, 1-H), 4.63 (d, *J* = 5.4 Hz, 1H, ), 5.92(d, *J* = 6.0 Hz, 1H), 7.10-7.54 (m, 4H, ArH). Anal calcd. for C_18_H_20_N_2_O_4_: C, 65.85; H, 6.10; N, 8.54. Found: C, 65.89; H, 6.21; N, 8.48.

7e: IR (KBr, cm^-1^) : 1645,1685,1710. MS m/z 344 (M^+^). ^1^H NMR (300 MHz, CDCl_3_) δ 1.20 (m, 1H, 2-H), 1.76 (m, 1H, 2-H), 2.86 (m, 1H, 3-H), 3.28 (s, 3H, NMe), 3.46 (s, 3H, NMe), 3.80 (s, 3H, OCH_3_), 4.02 (m, 1H, 1-H), 4.28 (m, 1H, 1-H), 4.52 (d, *J* = 5.5 Hz, 1H, ), 5.94 (d, *J* = 6.0 Hz, 1H), 7.05-7.52 (m, 4H, ArH). Anal calcd. for C_18_H_20_N_2_O_5_: C, 62.79; H, 5.81; N, 8.14. Found: C, 62.82; H, 5.89; N, 8.05.
